# Supplementary material for: let-7 miRNA and lin-46 mRNA are essential targets of the LIN28 RNA-binding protein in Caenorhabditis elegans developmental timing regulation
Source: PLoS Biol. 2026 Aug 4;24(8):e3003892. doi: 10.1371/journal.pbio.3003892 (PMC13436749; doi:10.1371/journal.pbio.3003892)
Supplement: Supplementary file 1 [file pbio.3003892.s001.pdf]

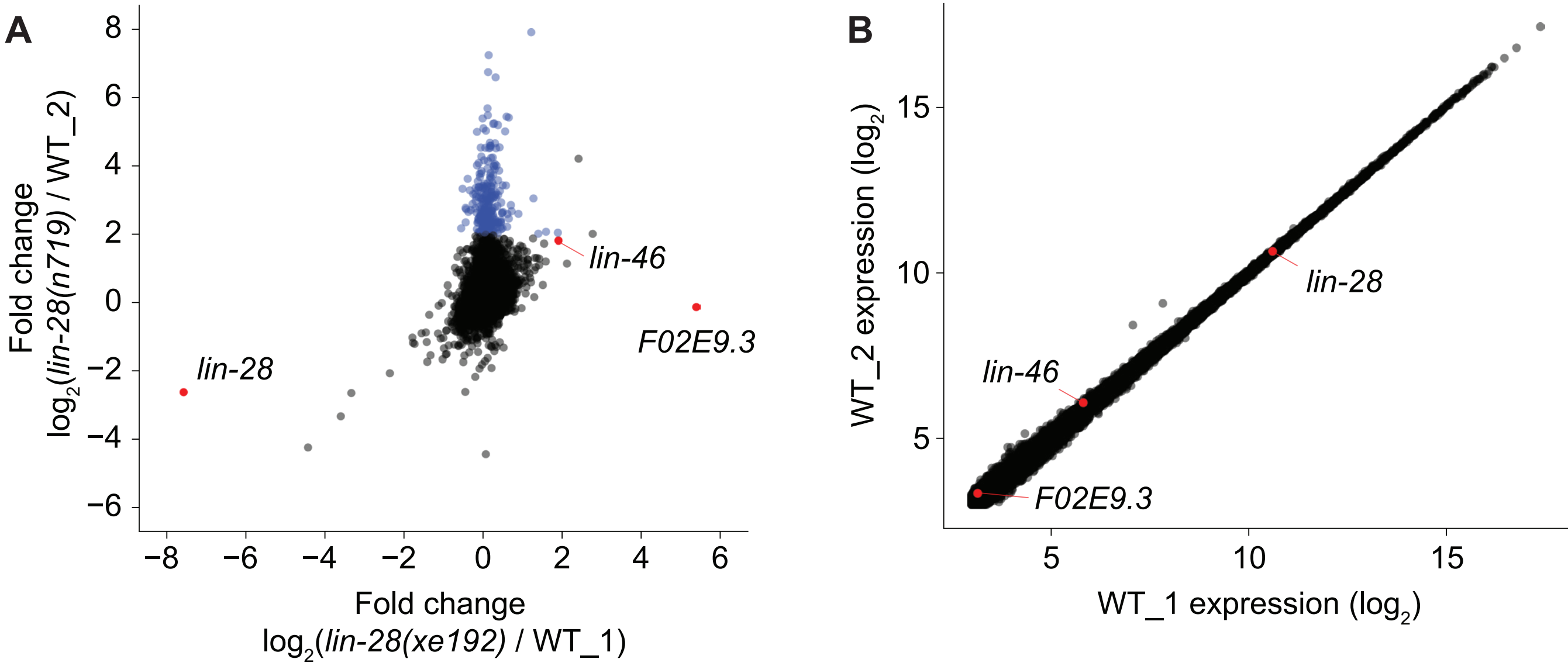

A
8
6
4
2
0
−2
−4
−6
Fold change
log2(lin-28(n719) / WT_2)
lin-46
F02E9.3
lin-28
−8
−6
−4
−2
0
2
4
6
Fold change
log2(lin-28(xe192) / WT_1)
B
15
10
5
WT_2 expression (log2)
lin-28
lin-46
F02E9.3
5
10
15
WT_1 expression (log2)
