# Supplementary material for: let-7 miRNA and lin-46 mRNA are essential targets of the LIN28 RNA-binding protein in Caenorhabditis elegans developmental timing regulation
Source: PLoS Biol. 2026 Aug 4;24(8):e3003892. doi: 10.1371/journal.pbio.3003892 (PMC13436749; doi:10.1371/journal.pbio.3003892)
Supplement: Supplementary file 2 [file pbio.3003892.s002.pdf]

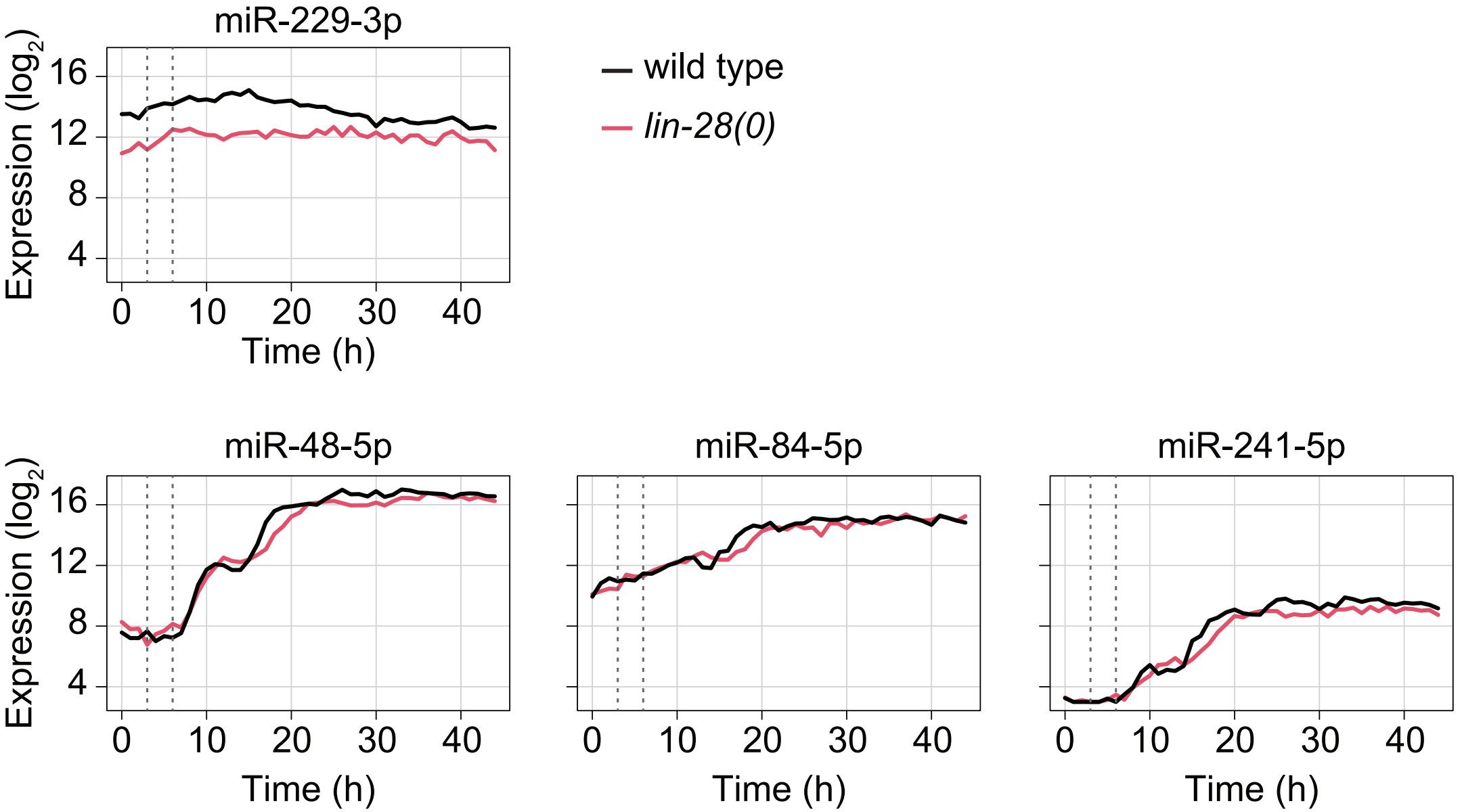

miR-229-3p
wild type
lin-28(0)
miR-48-5p
miR-84-5p
miR-241-5p
Expression (log2)
Time (h)
16
12
8
4
0
10
20
30
40
