# Supplementary material for: let-7 miRNA and lin-46 mRNA are essential targets of the LIN28 RNA-binding protein in Caenorhabditis elegans developmental timing regulation
Source: PLoS Biol. 2026 Aug 4;24(8):e3003892. doi: 10.1371/journal.pbio.3003892 (PMC13436749; doi:10.1371/journal.pbio.3003892)
Supplement: Supplementary file 3 [file pbio.3003892.s003.pdf]

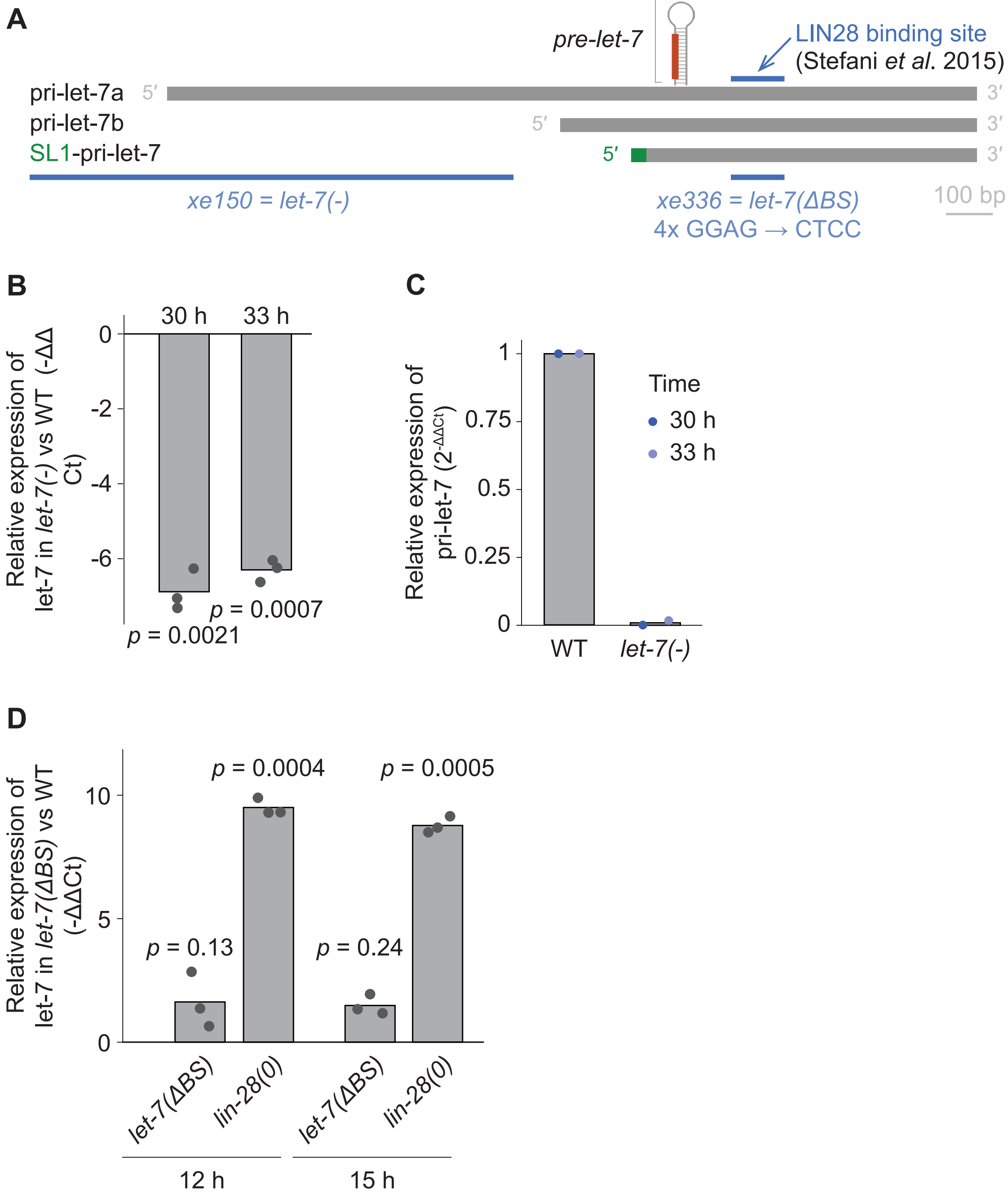
A
pre-let-7
LIN28 binding site
(Stefani et al. 2015)
pri-let-7a
pri-let-7b
SL1-pri-let-7
5′
3′
xe150 = let-7(-)
xe336 = let-7(ΔBS)
4x GGAG → CTCC
100 bp
B
Relative expression of let-7 in let-7(-) vs WT (-ΔΔ Ct)
30 h
33 h
0
-2
-4
-6
p = 0.0021
p = 0.0007
C
Relative expression of pri-let-7 (2-ΔΔCt)
1
0.75
0.5
0.25
0
Time
30 h
33 h
WT
let-7(-)
D
Relative expression of let-7 in let-7(ΔBS) vs WT (-ΔΔCt)
10
5
0
p = 0.13
p = 0.0004
p = 0.24
p = 0.0005
let-7(ΔBS)
lin-28(0)
let-7(ΔBS)
lin-28(0)
12 h
15 h
