# Supplementary material for: let-7 miRNA and lin-46 mRNA are essential targets of the LIN28 RNA-binding protein in Caenorhabditis elegans developmental timing regulation
Source: PLoS Biol. 2026 Aug 4;24(8):e3003892. doi: 10.1371/journal.pbio.3003892 (PMC13436749; doi:10.1371/journal.pbio.3003892)
Supplement: Supplementary file 4 [file pbio.3003892.s004.pdf]

Genotype	Condition	Alae appearance
wild type	M4 20°C	complete strong
lin-28(0)	M2 25°C	partial very weak
lin-28(0)	M3 25°C	partial weak
lin-28(0)	M3 20°C	complete strong (15%), complete weak (42.5%), partial weak (42.5%)
lin-28(0); let-7(-)	M4 20°C	weak patch (36%), partial weak (64%)
lin-46(0)	M4 20°C	complete strong (67%), strong gapped (33%)
lin-28(0); lin-46(0)	M4 20°C	complete strong (71%), complete partially weak (29%)
