# Supplementary material for: let-7 miRNA and lin-46 mRNA are essential targets of the LIN28 RNA-binding protein in Caenorhabditis elegans developmental timing regulation
Source: PLoS Biol. 2026 Aug 4;24(8):e3003892. doi: 10.1371/journal.pbio.3003892 (PMC13436749; doi:10.1371/journal.pbio.3003892)
Supplement: Supplementary file 6 [file pbio.3003892.s006.pdf]

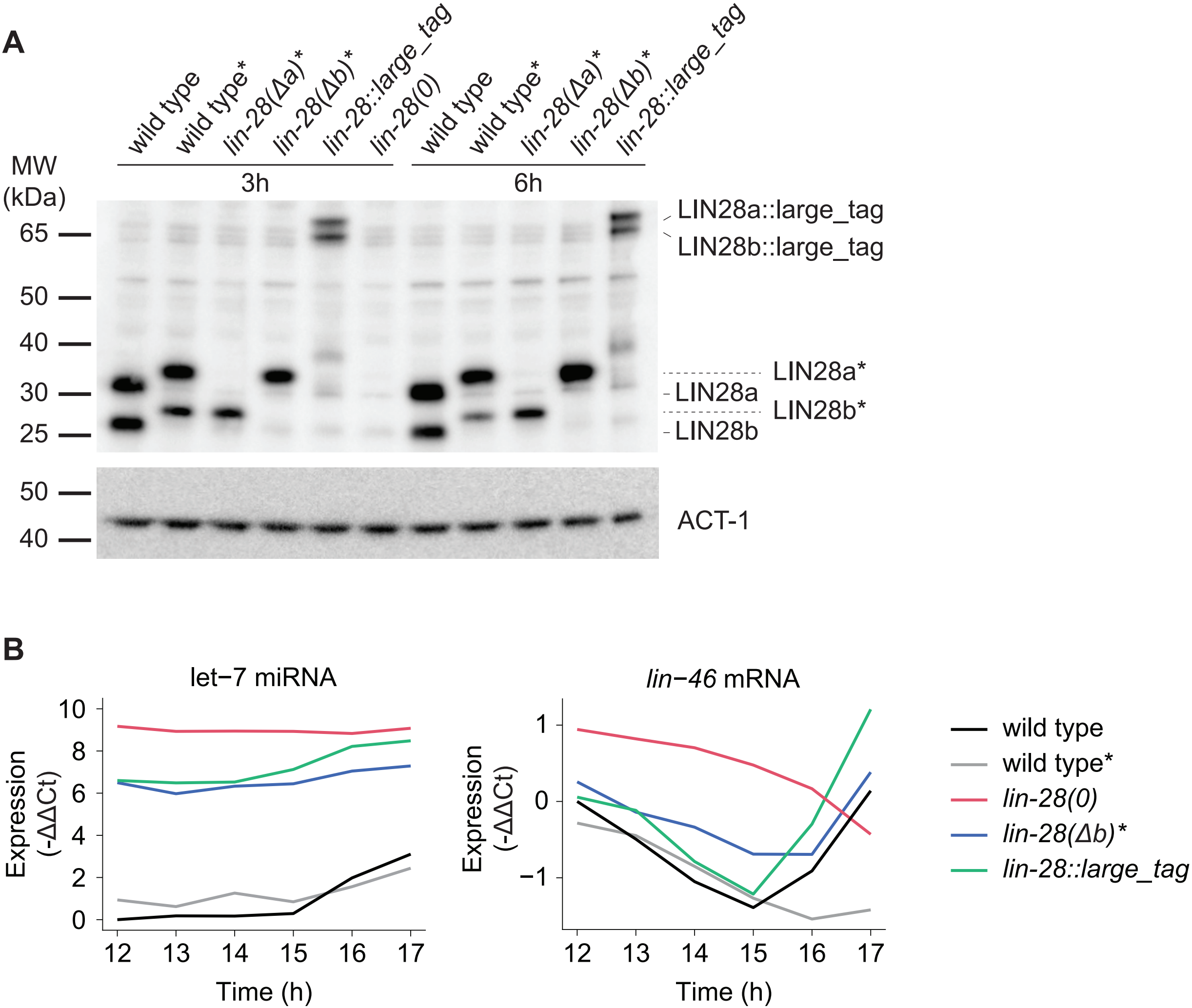

A
wild type
wild type*
lin-28(Δa)*
lin-28(Δb)*
lin-28::large_tag
lin-28(0)
wild type
wild type*
lin-28(Δa)*
lin-28(Δb)*
lin-28::large_tag
3h
6h
MW (kDa)
65
50
40
30
25
50
40
LIN28a::large_tag
LIN28b::large_tag
LIN28a*
LIN28a
LIN28b*
LIN28b
ACT-1
B
let−7 miRNA
lin−46 mRNA
Expression (-ΔΔCt)
Time (h)
wild type
wild type*
lin-28(0)
lin-28(Δb)*
lin-28::large_tag
