# Supplementary material for: let-7 miRNA and lin-46 mRNA are essential targets of the LIN28 RNA-binding protein in Caenorhabditis elegans developmental timing regulation
Source: PLoS Biol. 2026 Aug 4;24(8):e3003892. doi: 10.1371/journal.pbio.3003892 (PMC13436749; doi:10.1371/journal.pbio.3003892)

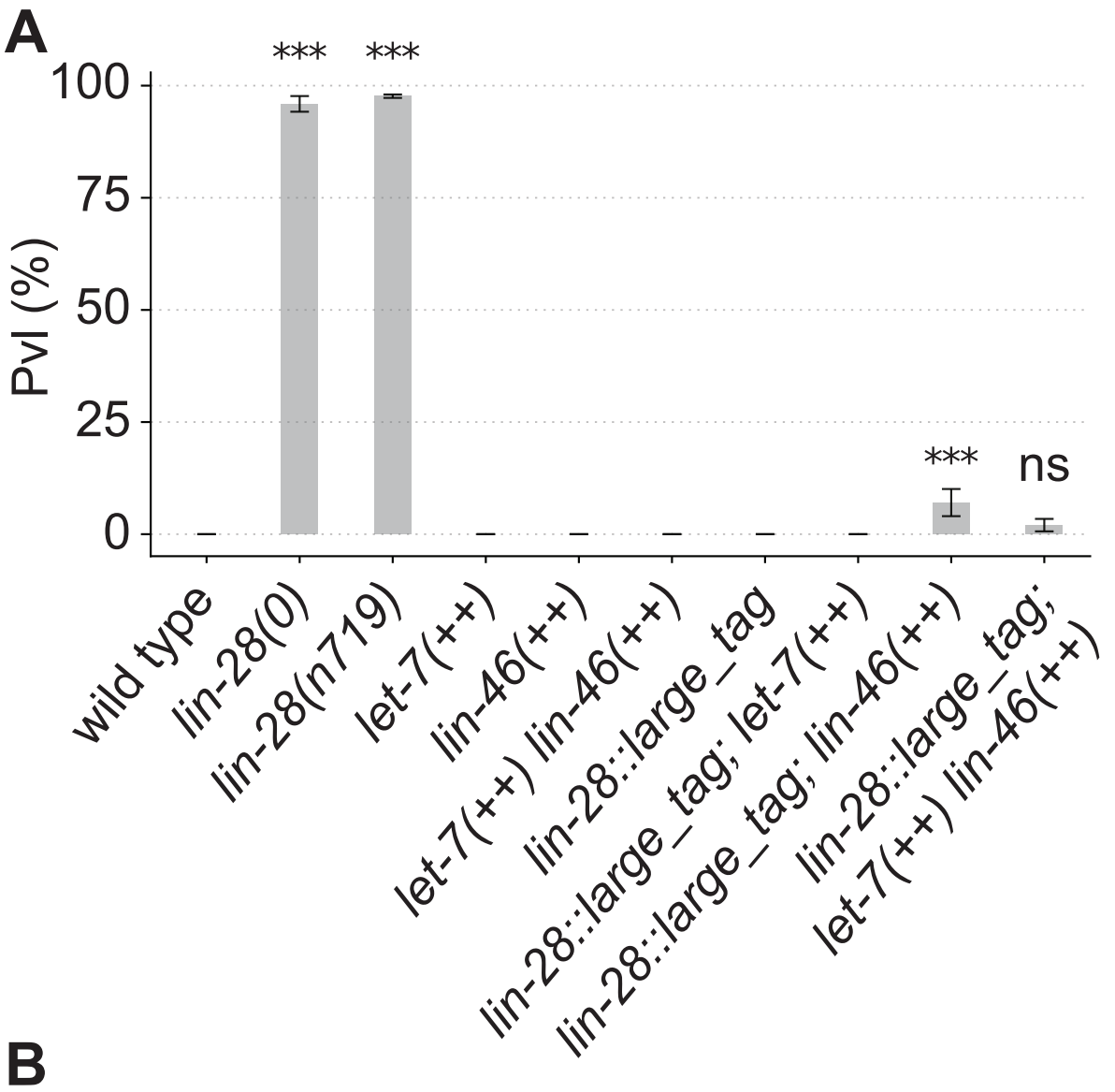

B

Genotype	% with any alae at M3 exit (n)
wild type	0 (19)
lin-28(0)	81 (16)
let-7(++)	0 (16)
lin-46(++)	0 (18)
let-7(++) lin-46(++)	0 (18)
lin-28::large_tag	0 (19)
lin-28::large_tag; let-7(++)	15 (20)
lin-28::large_tag; lin-46(++)	100 (19)
lin-28::large_tag; let-7(++) lin-46(++)	95 (19)

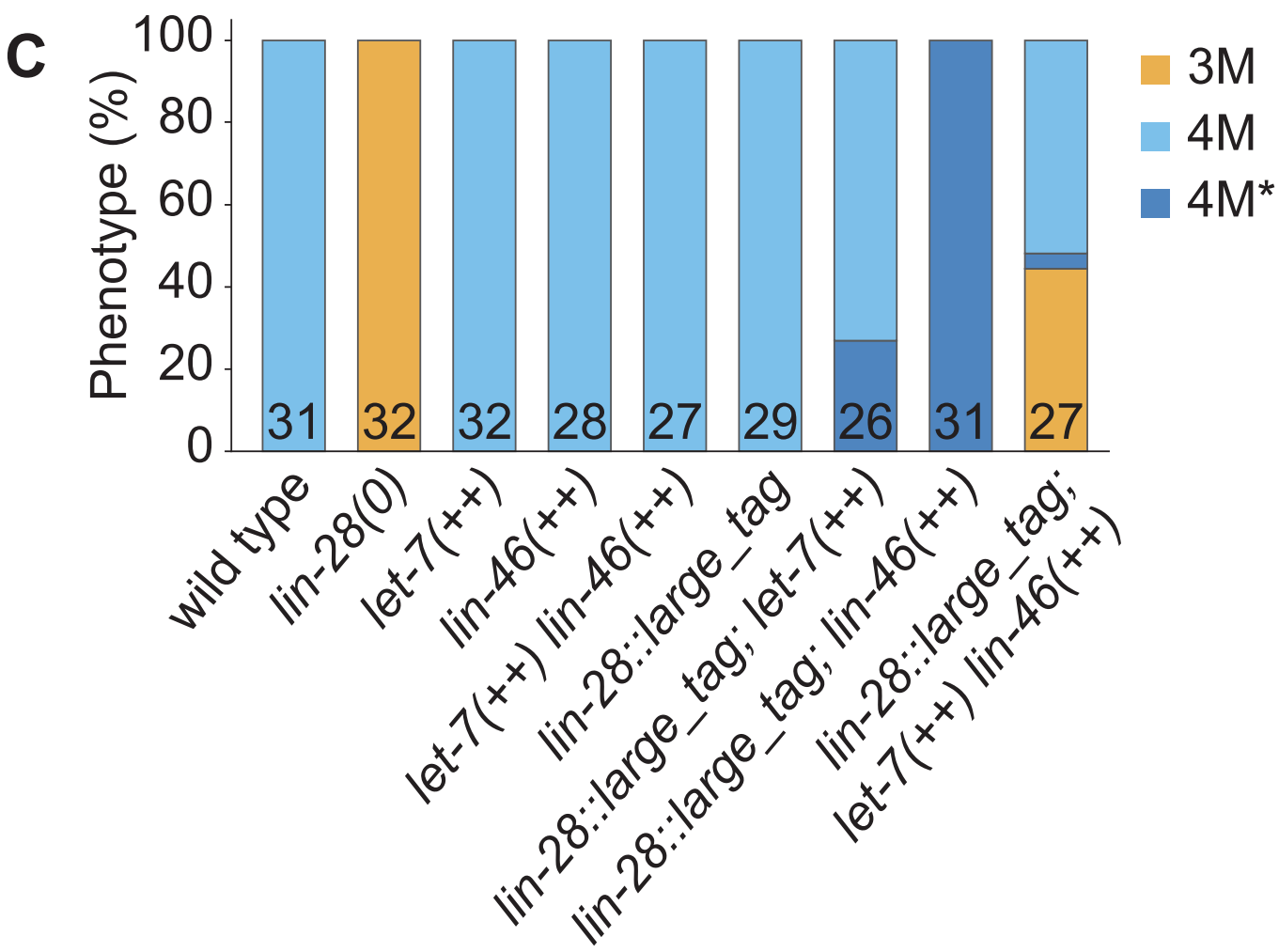

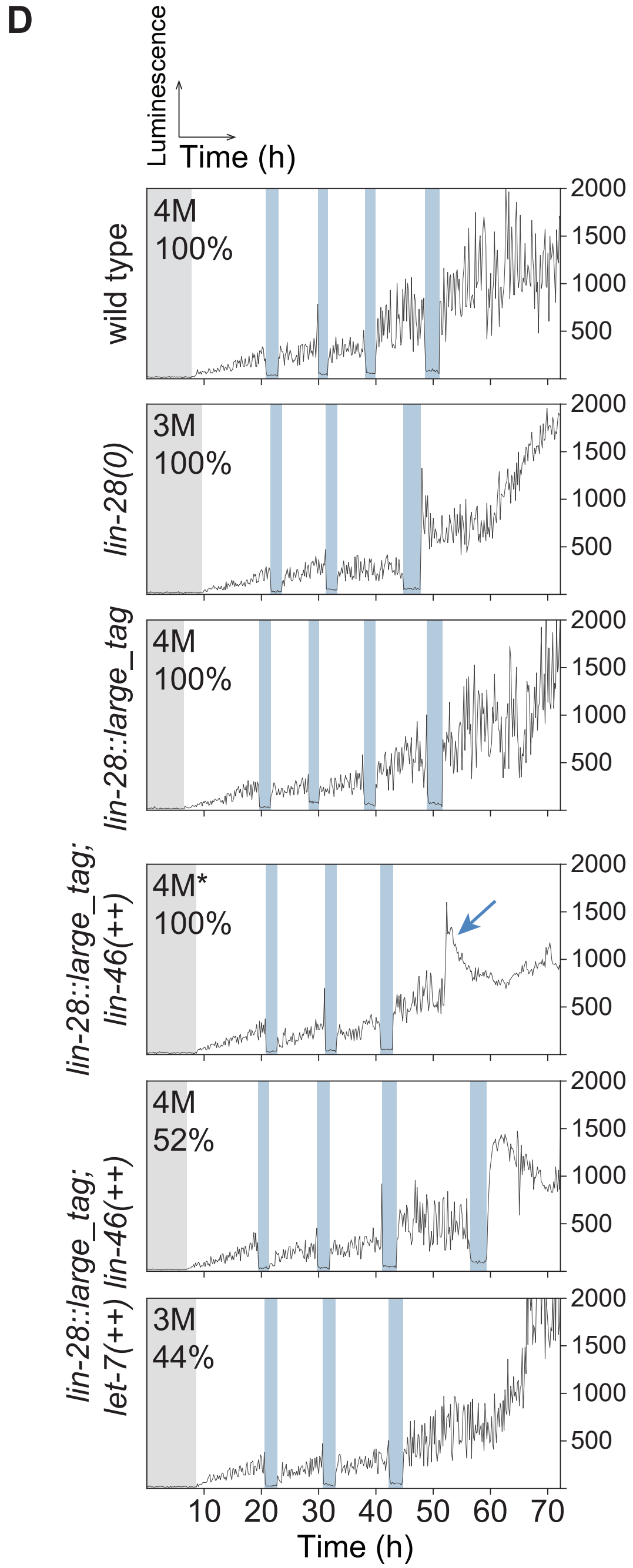

Supplement: Supplementary file 7 [file pbio.3003892.s007.pdf]
