# Supplementary material for: let-7 miRNA and lin-46 mRNA are essential targets of the LIN28 RNA-binding protein in Caenorhabditis elegans developmental timing regulation
Source: PLoS Biol. 2026 Aug 4;24(8):e3003892. doi: 10.1371/journal.pbio.3003892 (PMC13436749; doi:10.1371/journal.pbio.3003892)
Supplement: Supplementary file 8 [file pbio.3003892.s008.pdf]

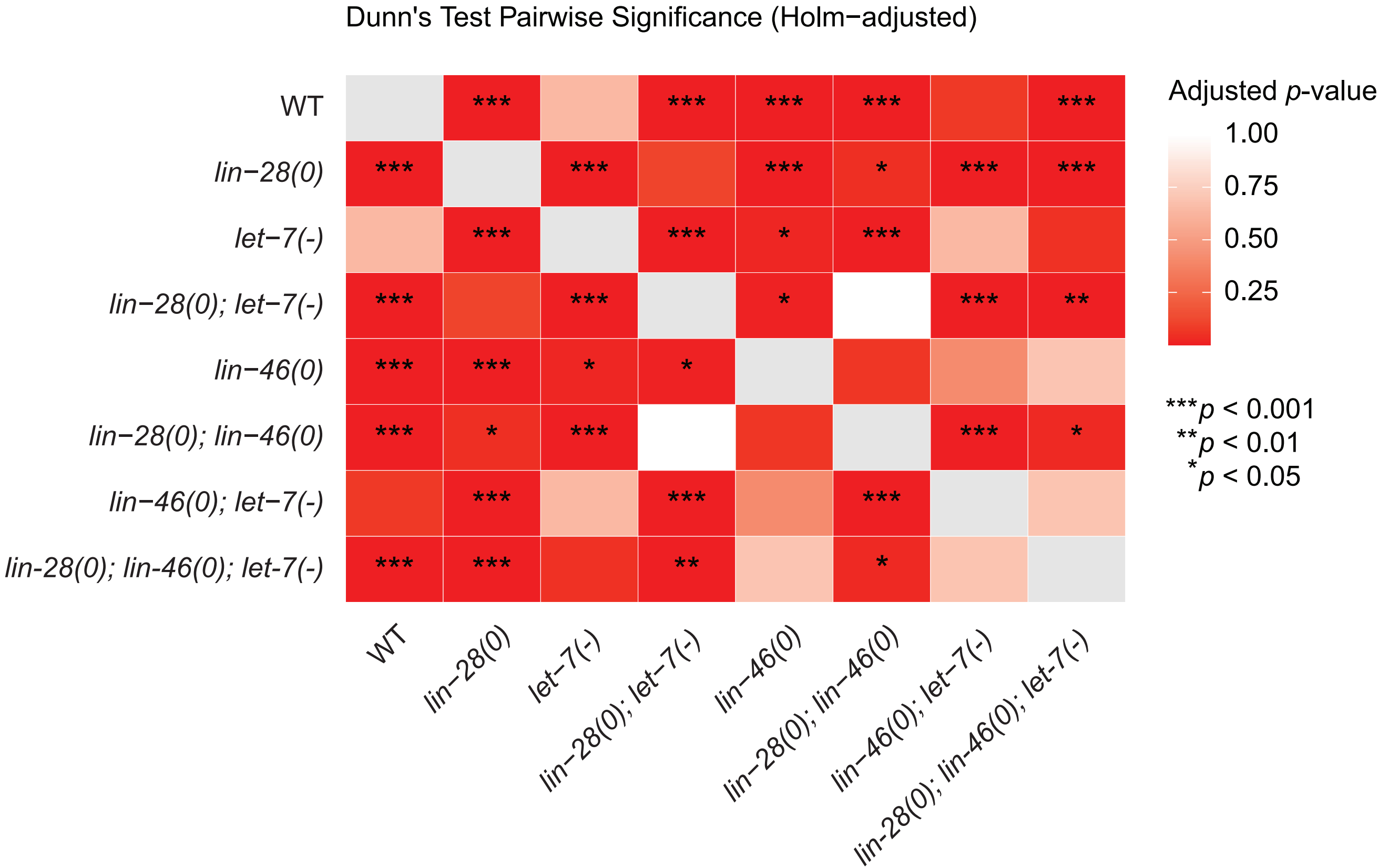
Dunn's Test Pairwise Significance (Holm−adjusted)
Adjusted p-value
1.00
0.75
0.50
0.25
***p < 0.001
**p < 0.01
*p < 0.05
WT
lin−28(0)
let−7(-)
lin−28(0); let−7(-)
lin−46(0)
lin−28(0); lin−46(0)
lin−46(0); let−7(-)
lin-28(0); lin-46(0); let-7(-)
