# Supplementary material for: let-7 miRNA and lin-46 mRNA are essential targets of the LIN28 RNA-binding protein in Caenorhabditis elegans developmental timing regulation
Source: PLoS Biol. 2026 Aug 4;24(8):e3003892. doi: 10.1371/journal.pbio.3003892 (PMC13436749; doi:10.1371/journal.pbio.3003892)

Fig. S6A: Western blot - Generation of *lin-28(Δb)* isoform-specific mutant

Original TIFF images were opened in Fiji (ImageJ), where display contrast was adjusted uniformly by setting the minimum and maximum intensity values to 900 and 3600 for LIN-28, and 550 and 1700 for ACT-1 detection, respectively. Images were then converted to 8-bit format and exported as TIFF files. The resulting images were subsequently opened in Adobe Illustrator for figure assembly and annotation.

anti-LIN-28 (GenScript, R13880)
Exposure 1s
Amersham Imager 680

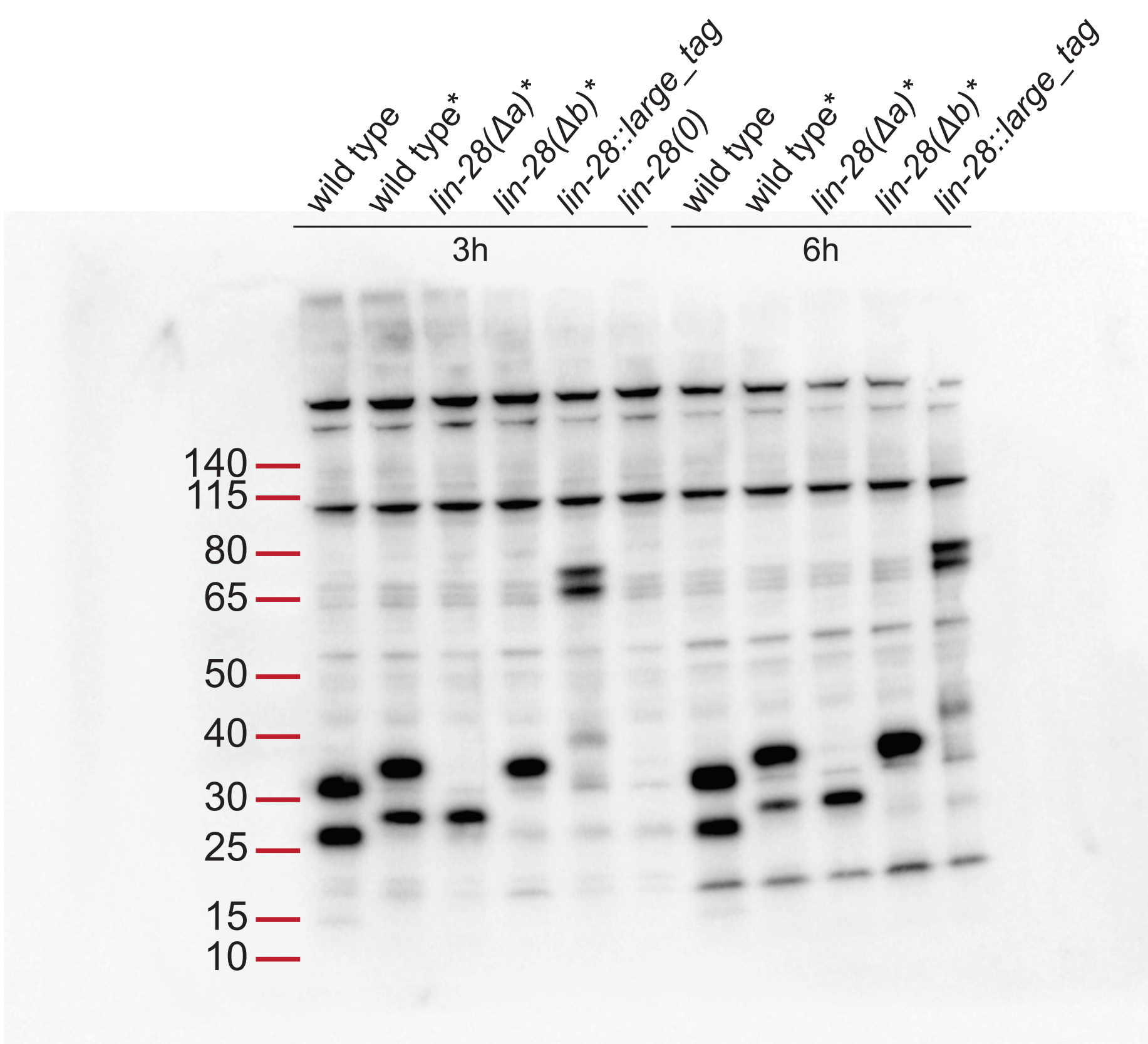

anti-ACT-1 (MAB1501)
Upon stripping
Exposure 5s
Amersham Imager 680

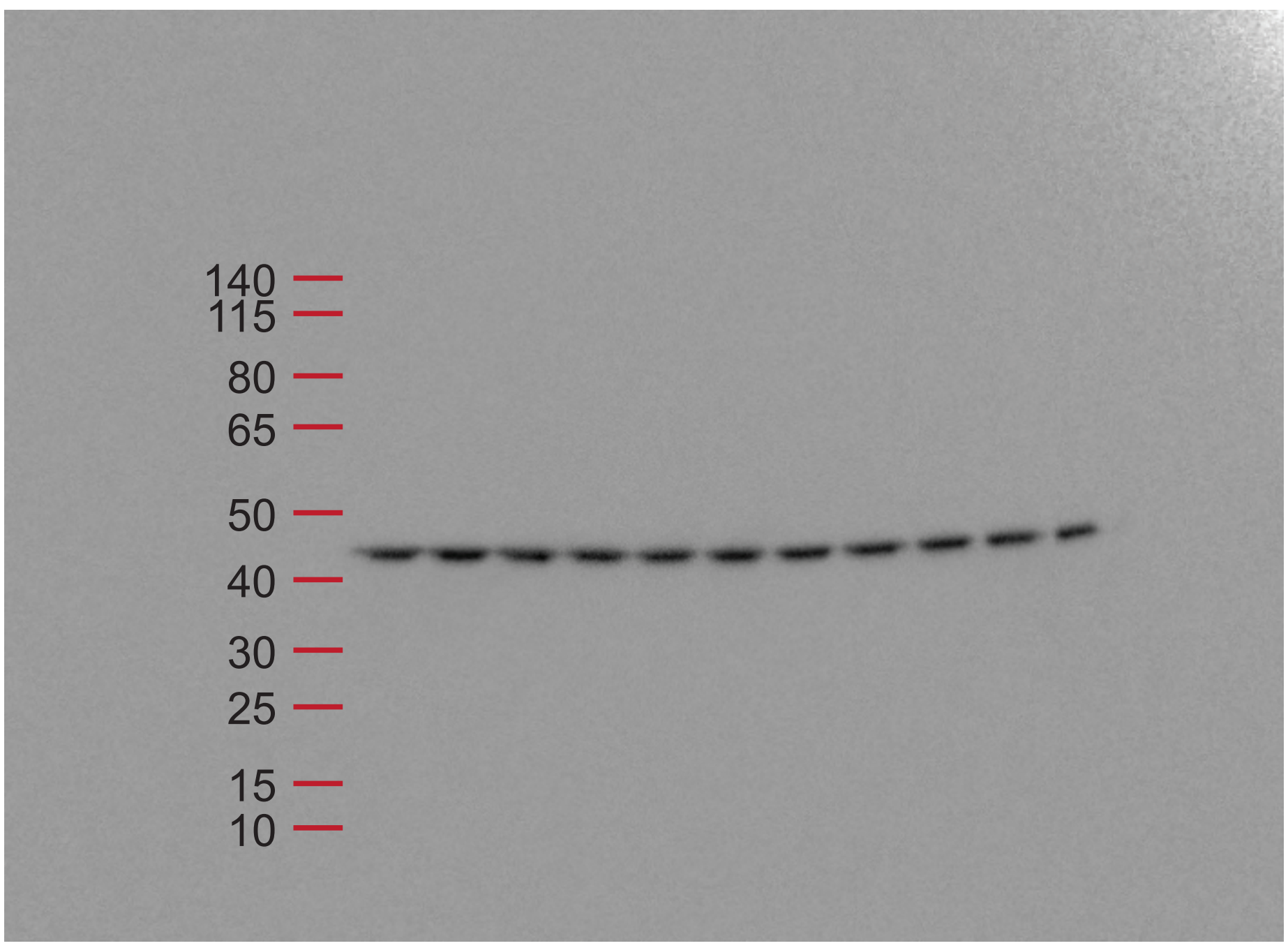

Supplement: Supplementary file 9 [file pbio.3003892.s009.pdf]
