# Supplementary material for: let-7 miRNA and lin-46 mRNA are essential targets of the LIN28 RNA-binding protein in Caenorhabditis elegans developmental timing regulation
Source: PLoS Biol. 2026 Aug 4;24(8):e3003892. doi: 10.1371/journal.pbio.3003892 (PMC13436749; doi:10.1371/journal.pbio.3003892)
Supplement: Supplementary file 11 [file pbio.3003892.s016.docx]

**Manuscript number:** RC-2026-03390

**Corresponding author(s):** Helge Großhans

*[Please use this template only if the submitted manuscript should be considered by the affiliate journal as a full revision in response to the points raised by the reviewers.*

*If you wish to submit a preliminary revision with a revision plan, please use our "*[*Revision Plan*](https://docs.google.com/document/u/0/d/1HHp62Tfeb44nd3Gu_3ChB0b8gmMdEog7GjZcZFwHSiQ/edit)*" template.* ***It is important to use the appropriate template to clearly inform the editors of your intentions.****]*

## 1. General Statements [optional]

*This section is optional. Insert here any general statements you wish to make about the goal of the study or about the reviews.*

Please note that line numbers have changed in the revised manuscript, which no longer contains embedded figures and legends; all new line numbers refer to the manuscript without tracked changes.

## 2. Point-by-point description of the revisions

*This section is mandatory.* *Please insert a point-by-point reply describing the revisions that were already carried out and included in the transferred manuscript.*

------------------------------------------------------------------------------ 
Reviewer #1 (Evidence, reproducibility and clarity (Required)): 

This interesting paper used the C. elegans system to investigate the molecular mechanisms by which the conserved RNA binding protein LIN-28 regulates development. In C. elegans, lin-28 promotes early cell fate in lateral hypodermal seam cells and vulval precursors as part of the heterochronic pathway. Prior work has implicated heterochronic genes lin-46 and let-7 as direct targets of LIN-28. This manuscript uses genetic and genomic approaches to argue that these two genes can explain all developmental phenotypes in lin-28 mutants. 

The key findings from this manuscript are as follows: 
• The authors used a combination of RNA-seq and RIP-seq to identify transcripts and microRNAs bound by LIN-28 whose expression changes in a lin-28(0) mutant. Surprisingly, they found only one mRNA, lin-46, and one microRNA, let-7, that fit both criteria. 
• The authors used genetic epistasis experiments with both loss-of-function and gain-of-function mutants to argue that lin-46 and let-7 together account for all lin-28(0) phenotypes. 
• The authors used precise, endogenous mutations to provide additional insight into LIN-28 binding sites on these targets. Specifically, they found that the lin-46 site only partially accounts for its regulation by lin-28, and that the proposed binding sites on the let-7 primary transcript did not influence let-7 levels. 
• The authors identified a role for lin-28 in the duration of larval stages.

We thank the reviewer for their careful evaluation and strong endorsement of our work. We have addressed their remaining criticisms as detailed below.

Major comments: 

1. The text and figures are well-written and clear, and in general the key conclusions are convincing. However, there is one point that we think must be addressed to strengthen the claim that lin-46 and let-7 are the only two relevant targets for LIN-28. This point concerns the question of whether let-7(xe150) is truly a null allele. 

As we see it, the argument that lin-46 and let-7 are the two key LIN-28 targets comes from the following data. First, lin-46 and let-7 are the only two RNAs that are both bound by LIN-28 and whose levels increase significantly in lin-28(0) mutants. The data here are convincing, but do not preclude regulation of mRNA translation that does not affect mRNA stability, which is common among RNA binding proteins. In other words, there may be more relevant LIN-28 targets that are regulated at the translational level and thus would not be identified by RNA-seq data. 

Second, weak overexpression of lin-46 and let-7 in a lin-28 hypomorphic background recapitulates lin-28(0) phenotypes. These data are consistent with lin-46 and let-7 being the sole targets, but they are also consistent with additional targets being upregulated in the lin-28 hypomorphic background and contributing to the phenotype. 

Therefore, the critical data supporting the argument that lin-46 and let-7 are the sole relevant LIN-28 targets is the genetic epistasis data using loss-of-function alleles to demonstrate that a lin-46; let-7 double mutant is epistatic to loss of lin-28 across multiple phenotypes. However, these experiments are only interpretable when null alleles are used. A strength of this paper is that the alleles for the protein-coding genes are clear null alleles: deletions of the entire coding regions of lin-28 and lin-46. In contrast, the xe150 allele termed "let-7(0)" is a promoter mutation that retains the let-7 sequence. While the authors show qPCR data indicating that expression of let-7 is greatly reduced in this background, very low expression is not the same as a null, which implies zero gene product. 

Can the authors provide more evidence that the let-7(xe150) allele is indeed a null? Is it clear that let-7 cannot be transcribed with this mutation? Without that evidence, key epistasis experiments should be repeated with a null allele in order to be able to interpret these experiments.

We provide the requested evidence for pri-let-7, which appears consistent with our initial assessment below, but would also like to politely disagree with the notion that epistasis analysis is fully dependent on null alleles. Use of hypomorph mutations does put certain limits on interpretation of such analyses, but these are not relevant to our conclusions.

Specifically, if the downstream gene is non-null (as the reviewer hypothetically considers in our scenario for *let-7*), the specific risk is that the compound mutant animals may not exhibit a full null mutant phenotype for that allele, and that this milder phenotype may potentially be “masked” by a more severe null phenotype of the upstream gene. Neither is true in the current scenario: first, *let-7(xe150)* animals exhibit the full scope and penetrance of phenotypes previously described for *let-7(mn112)*, the reference allele. Second, the *lin-28; lin-46; let-7* triple mutant animals exhibit exactly those phenotypes that are also seen for *let-7(xe150)* and *let-7(mn112)* single mutant alleles. Hence, we can conclude that even under the assumption that *let-7(xe150)* were a severe hypomorph, not a null, our conclusions would hold.

We do not consider use of *let-7(mn112)*, the only reported “null” allele (Reinhart et al., Nature 2000), a suitable alternative since it additionally carries a linked *unc-3* mutation that causes uncoordinated movements and thereby defects in moving on bacterial lawns and feeding. It also might not qualify as a null allele by the reviewer’s criterion since it continues to transcribe pri-let-7 (Bracht et al., RNA 2004). The levels of mature let-7 potentially present in that strain are unknown, since it was only examined by Northern Blot (Reinhart et al., Nature 2000, Bracht et al., RNA 2004), which typically has a much smaller dynamic range than the RT-qPCR assay used by us.

At any rate, we have now determined pri-let-7 levels at 30 h and 33 h of development at 25°C, when mature let-7 levels are at their peak in wild-type animals (Nahar et al., Nucleic Acids Res, 2024). At 30 h, the signal in *let-7(xe150)* is < 1/1000 of wild-type, at 33 h no signal is detectable at all. (We used Ct=40 as the lower level of detection to calculate the level of reduction in Fig. S3C.) Hence, although we agree that, retrospectively, full deletion of the locus would have made the results even less ambiguous, we believe that we can consider this allele as a null. At any rate, we now explicitly acknowledge in the text (ll. 183-186) that it remains formally possible that *xe150* is a very strong hypomorph.

2. A smaller concern regarding the genetic epistasis data presented in Figure 3A is that the precocious phenotypes are mostly addressed at 25˚C and not at 20˚C. The data presented indicate that the lin-28(0) precocious alae phenotype is stronger at 20˚C than 25˚C, making the suppression of precocious phenotypes more difficult to interpret when only 25˚C is shown. For the one strain where data at both 20˚C and 25˚C are presented (lin-28; let-7), complete suppression is observed at 25˚C but partial suppression was observed at 20˚C. Thus, it seems possible that other strains that appear to completely suppress the lin-28(0) phenotype at 25˚C may not do so at 20˚C. Furthermore, the phenotypes at M4 are only presented at 20˚C. Adding M3 data at 20˚C for strains containing lin-46 would strengthen the argument that the precocious phenotypes are indeed fully suppressed in those backgrounds. In addition, the statement on line 307-309 should be adjusted because it now claims that lin-28(0); let-7(0) double mutants do not exhibit any alae after the third molt, whereas Figure 3A shows that 6% of lin-28; let-7 animals have alae at M3 at 20˚C. It seems fair to say that there is strong suppression at both temperatures, but not quite 'full' suppression.

We agree with the reviewer’s criticism and have performed the requested experiments at 20˚C. These reveal that suppression is equally potent at 20˚C as at 25˚C, validating our earlier conclusions. Specifically, we find that 98% of *lin-28(0)* animals have at least some alae at M3 exit, whereas none of the other examined strains has any alae at that time (that is *lin-28; let-7* double, *lin-28; lin-46* double and the triple mutant.) These data are shown in the updated Fig. 3A.

We have adjusted our wording of the statement previously made in ll. 307-309 (now ll. 211-213) to read: “The precocious alae synthesis is strongly suppressed by loss of either *lin-46* or *let-7*, with no or very rare alae in *lin-28(0); lin-46(0)* and *lin-28(0); let-7(0)* double mutant animals after the third molt.”

3. The data and methods are thorough and clearly described. There is one small exception that should be clarified. We could not find detailed information about the xeEx365 array. Can more information be provided? The reader should be able to reconstruct the exact sequence of the let-7 construct.

We apologize for not clarifying that the array was from a previous study, which has now been referenced (Ecsedi et al., Dev Cell 2015). We do point out that according to that manuscript, the array should drive *let-7* from the *tbb-1* promoter, but subsequently, based on additional genotyping data, was redesignated *let-7p::let-7* (Azzi et al., eLife 2020). Unfortunately, we could not trace back where that apparent mix-up occurred. However, since the array is merely used as a balancer for strain maintenance/propagation, whereas all reported results are in the absence of the array, the promoter identity has no impact on our conclusions.

Minor comments 
1. Lines 101-106: does a "genetically clean" background mean without chemical mutagenesis? Was the lin-28(n719) allele backcrossed?

Yes, we have now clarified in the main text (ll. 107-108) that the *lin-28(xe192)* deletion mutation was introduced in a targeted manner, by CRISPR-Cas9-based genome engineering, as described in the Methods.

The *lin-28(n719)* allele was obtained by crossing it out of a published *lin-28(n719); let-7(mn112) unc-3(e151)* strain (from Reinhart et al., 2000), as now clarified in the legend to Fig. S1. No further outcrosses were done. We did not find any information on the outcrossing status of the starting strain in the original paper.

2. Line 96: "6 h at 25˚C after plating". What was plated? From reading the methods, it appears likely to be starved L1s (as opposed to embryos). Is this correct?

Yes, this is correct and we have updated the text accordingly (l. 98).

3. Figure S1: Since each lin-28 mutant was compared to independent wild-type samples, how do the two wild-type samples compare to each other? That information will help the reader understand whether any of the differences between lin-28(n719) and lin-28(xe192) could be because of slight variations in timing or other technical differences if the samples were not all collected in parallel.

The samples were indeed collected in parallel, with separate wild-type replicates. We have now included an additional figure panel, Fig. S1B, showing that the wild-type samples correlate very highly and thus do not drive the variation seen with the *lin-28* mutant strains.

4. Figure 3B: Including examples of strong and weak alae is useful. Can more explanation be provided about the context for these different types of alae? Are the M2 alae always weak and the M3 alae always strong?

This aligns with a question by Reviewer #3 (Minor Comment #4). To address it, we now provide a new Supplementary Figure Fig. S4 providing further information on alae quality. Interestingly, although M3 alae are generally of “better quality” (stronger, more complete), only a small fraction of *lin-28(0)* animals exhibits complete and strong alae at M3.

5. Figure S5: lin-28(∆a) is presented in the figure but never discussed in the text. Can a short explanation be provided?

As we now clarify in the figure legend, *lin-28(∆a)* is not characterized further in the present study. We included it because we wished to avoid splicing of the blots and considered it an additional, useful control.

6. Lines 671-673 and 803-808: The authors note a discrepancy between their data for mutation of the putative LIN-28 binding site and previously published data (Ilbay & Ambros 2021). Right now, this is described in the Methods section. Consider making a supplemental figure for this information so that it can be described more completely, including the details of the newly generated strains. Also note that it is unlikely that the maIs105 transgene accounts for all of the differences observed, because the Ilbay and Ambros paper also includes lin-46 5'UTR mutations made without maIs105: those in the background of tagged LIN-46. In Figure 3B of the Ilbay and Ambros paper, lin-46(ma389 ma459) appears to display precocious vulval development as well as precocious LIN-46 expression, and it does not appear that maIs105 was present in this strain.

We have meanwhile pursued this a bit further and find, through genome-resequencing, that the original VT3855 (*lin-46(ma467); maIs105)* strain from Ilbay et al., Cell Rep 2021 additionally contains a ~7.6-kb deletion very close to *lin-46* (Katic et al., in preparation) as shown in Reviewer Figure #1 below. Together with the fact that we cannot outcross the *maIs105* array from that strain although we could easily cross in the array into our remade *lin-46* allele, we believe that VT3855 has likely additional unaccounted genomic variation. At any rate, it is clear that the reported Pvl phenotype cannot be reliably assigned to the intended *lin-46* mutation. It remains to be seen whether this is a one-off issue or reflects a more systematic issue of sgRNAs or strain used for injection in Ilbay et al.; we have flagged our findings to the corresponding author of that study.

**Reviewer Figure 1:** Genome Browser View of the region around the lin-46 locus in VT3855 (top) and an unrelated strain (middle). Bottom: RefSeq gene annotation, *lin-46* boxed in red. Katic et al., in preparation.

We provide the phenotypic characterization data requested by the reviewer for their inspection as Reviewer Figure 2 below. However, we further learned from Dr. Ambros that the actual mutation of *ma467* differs by an additional nucleotide insertion from what they had intended and published. In other words, VT3855 deviates in at least three ways from the intended genotype. Since it is thus impossible to link its Pvl phenotype causally to the intended *lin-46* mutation, we see no benefit in including further phenotypic characterization of VT3855 as a figure in our manuscript. We find that the combination of flagging the general issues in the Results and pointing to the Methods for further detail (which we have updated with information on the above issues, ll. 537-544) is preferred.

**Reviewer Figure 2:** A newly generated *lin-46gf* allele, *lin-46(xe381)*, does not recapitulate the Pvl phenotype of VT3855 (*lin-46(ma467); maIs105*, Ilbay et al., Cell Rep 2021).

**A**: Using CRISPR/Cas9, we regenerated the *ma467* allele described in Ilbay et al. to produce HW3659: *lin-46(xe381)*. (We note that in the meantime we learnt from Dr. Ambros that ma467 has an additional nucleotide insertion next to the deletion that had not been previously reported.) Animals were grown at 20°C and examined at 52 h of development, when they had reached adulthood. N = 3 technical replicates, with n > 110 animals scored per genotype. Error bars represent standard deviation. Significant differences were determined by an unpaired two-sided t-test (ns: not significant; ***p < 0.001).

**B**: To test to which extent the *maIs105* array contributes to the Pvl phenotype, we crossed the array (strain VT1367: *maIs105* V) into our regenerated strain (HW3659: *lin-46(xe381)* V). This was easily feasible (2/29 GFP homozygous F2 animals segregated *lin-46(xe381)*) and did not result in a Pvl phenotype. Animals were grown at 20°C and examined at 72 h of development. Strains used: VT3855: *lin-46(ma467) maIs105* (n=134), IFM448: *maIs105* and IFM447 *lin-46(xe381) maIs105* (n=92). Note that IFM448 and IFM 447 are siblings, obtained by crossing VT1367 with HW3659 and selecting *lin-46(+)* and *lin-46(xe381)* progeny, respectively, carrying *maIs105*. Katic et al., in preparation.

Finally, although the reviewer is correct that *lin-46(ma398 ma459)* mutant animals were reported to cause a Pvl phenotype despite lacking the *maIs105* array (and our new data indicate that *maIs105* may not or only partially account for the Pvl phenotype), closer examination of the Key Resources Table from Ilbay et al. revealed a different issue for this strain: It is not only modified in the *lin-46* locus, but also expresses a GFP-tagged LIN-28. This tag compromises LIN-28 function as subsequently described the same authors (Nelson & Ambros, *bioRxiv* 2025, <https://doi.org/10.1101/2025.11.13.688268>). Hence, a Pvl phenotype in this strain likely reflects that sensitization, in agreement with our finding that enhanced expression of *lin-46* in the *lin-28(Δb)* background results in a Pvl phenotype. We were unable to obtain *lin-46* mutant lines with a Pvl phenotype and without further genetic complications (arrays, tagged *lin-28*) from the authors. Taken together, we conclude that a Pvl phenotype in a clean *lin-46gf* mutant awaits demonstration.

Reviewer #1 (Significance (Required)): 

The LIN-28 RNA-binding protein was first discovered in C. elegans for its role in promoting early cell fate as part of the heterochronic pathway. Interestingly, later work showed that this role is conserved in mammals and that part of the molecular pathway is also conserved. In both mammals and C. elegans, LIN-28 regulates the biogenesis of the let-7 microRNA, which promotes differentiation in both systems. Additionally, LIN-28 can also regulate target mRNAs in mammals and worms. Thus, molecular insights concerning LIN-28 are of interest to a broad range of scientists studying development, stem cells, and RNA biology. The major advance in this work is to define the role for lin-28 in regulating development on a molecular level by using modern genomic tools combined with classical genetic epistasis to describe the regulation of lin-46 and let-7 by lin-28. 

This paper builds on prior work investigating lin-28 and implicating lin-46 and let-7 as molecular targets. Briefly, lin-28 was one of the founding members of the heterochronic pathway (Ambros and Horvitz 1984). lin-46 was discovered as a gene that exhibited strong mutual suppression with lin-28 (Pepper et al. 2004). The idea that lin-28 has two separable activities was first described in Vadla et al. (2012). Vadla et al. also demonstrated that let-7 and not other microRNAs within the let-7-family contributed to lin-28 phenotypes. HITS-CLIP identified hundreds of mRNAs bound to LIN-28 and identified binding to both lin-46 mRNA and pri-let-7 (Stefani et al. 2015). Finally, prior work found that lin-28 regulates expression of the LIN-46 protein but not mRNA levels via the 5'UTR, and that mutations within the lin-46 5'UTR recapitulate lin-28(-) phenotypes (Ilbay and Ambros 2021). The current work under review confirms and extends much of this prior work, including the major role played by lin-46 and let-7 as the key LIN-28 targets. Furthermore, the authors identified some discrepancies with prior work, particularly with respect LIN-28 binding sites. 

This paper is likely to be of interest to a broad range of developmental biologists. It represents a conceptual advance and introduces strains and datasets that will be useful in the field. 

We again thank the reviewer for pointing out the conceptual advances and broad relevance of our work.

Reviewer #2 (Evidence, reproducibility and clarity (Required)): 

Summary: 
The manuscript by Brunner et al., investigates the molecular and biologically relevant targets of the LIN-28 RNA binding protein in C. elegans. While LIN-28 has been shown to binds thousands of RNAs, including the let-7 miRNA primary transcript, the regulatory role and biological importance of this extensive network has been unclear. Here the authors use molecular approaches to investigate the RNAs bound and regulated by LIN-28 and genetic experiments to determine which targets are important for C. elegans development and viability. Remarkably, only two genes, lin-46 and let-7, emerged as functionally essential targets. It was previously reported that the lin-46 mRNA is bound and translationally repressed by LIN-28 protein and processing of the let-7 primary transcript is blocked by LIN-28. Using careful and thorough molecular and phenotypic assays of genetic mutants, the authors now convincingly show that lin-46 and let-7 are the only relevant targets of LIN-28, at least under standard growth conditions. Furthermore, they reveal a new defect, slowed progression through larval stages, caused by loss of lin-28 and regulation of its targets. Additionally, this work expands our understanding of lin-46 regulation by LIN-28, showing that RNA destabilization is involved as well as likely more binding sites than the previously identified site in the 5'UTR. They also directly tested the predicted LIN-28 binding site in the let-7 primary transcript and found that its removal is not sufficient to reproduce the level of mis-regulation seen in the lin-28 loss of function strain. While the authors do not attempt to map the presumably additional LIN-28 regulatory sites in lin-46 or let-7 these studies do demonstrate that recognition of these targets by LIN-28 is not solved. Overall, the experiments are rigorous, well controlled and the results support the conclusions. The paper is scholarly and clearly written with attention to referencing prior studies and limitations of the study. 
Major Comments: 
None 

Minor Comments: 
None 


Reviewer #2 (Significance (Required)): 

This paper is significant because it deeply investigates the biologically important targets of the well conserved LIN-28 RNA binding protein. It provides a conceptual advance that pinpoints key genes that require regulation by LIN-28, despite its extensive binding repertoire. While it is surprising that only 2 targets emerged as relevant for the role of lin-28 in regulating the identity and pace of larval development, this finding may inform on the function of LIN28 in mammalian systems. Regulation of let-7 biogenesis is a conserved function of LIN28 but a consistent role in regulating the thousands mRNAs it binds in mammalian cells has been lacking. As mammalian LIN28 has roles in controlling puberty onset and cancer development, a better understanding of its biologically relevant targets will be of interest to a broad audience in the RNA and development fields. 

Amy Pasquinelli, PhD 

We thank Dr. Pasquinelli for her careful and positive evaluation of our work and its relevance to the scientific community.

Reviewer #3 (Evidence, reproducibility and clarity (Required)): 

Summary: In this manuscript, Brunner and colleagues dissect the contribution of LIN-28 targets to the overt phenotypes observed in LIN-28 loss of function animals. By generating new lin-28 alleles and performing RNA immunoprecipitation and RNA-seq, they demonstrate that very few mRNAs are altered in expression level by apparent LIN-28 binding. In fact, the only significantly deregulated mRNA outside the LIN-28 locus is a known target, lin-46. Among miRNAs, only the known target let-7 is upregulated. The authors perform careful epistasis tests, scoring each aspect of the lin-28 loss of function phenotype. Whereas previous results showed only partial or mutual suppression of these phenotypes by let-7 or let-46, the authors show that the three scored phenotypes were all completely suppressed in backgrounds mutated for both of these targets (now displaying the strongly retarded let-7 loss of function phenotype in the triple mutant). These results support a model wherein let-7 and lin-46 act redundantly downstream of lin-28 as the only targets driving the precocious lin-28 loss of function phenotypes. The authors further attempt to derepress let-7 and lin-46 from lin-28-mediated regulation by precise editing of LIN-28 binding sites in these transcripts, demonstrating no or partial derepression of the targets, respectively. Therefore the proposed binding sites in let-7 and lin-46 likely function redundantly with other motifs to enable LIN-28 binding. Nonetheless, the authors were able to use the partially derepressed lin-46 allele along with overexpression of let-7 to recapitulate lin-28 loss of function phenotypes in two lin-28 hypomorphic backgrounds. Finally, the authors demonstrate a new aspect of the lin-28 null phenotype that is similarly dependent on let-7 and lin-46: increased duration of larval stages. The authors speculate that this decrease in "tempo" is also a reflection of precocious development since the final larval stage in wild type is also slower than others. 

Generally, the conclusions in the manuscript are well supported by the experiments. 
A few very minor changes to the presentation are suggested.

We thank the reviewer for their careful and positive evaluation of our work. We have addressed their remaining minor comments as detailed below.

Minor Comments: 
1) Lines 215-219: "This suggests either that LIN28 acts preferentially through regulatory mechanisms other than RNA destabilization, or that most binding events be functionally dispensable." 
This sentence is perhaps too dense to convey everything that is meant here and is a little confusing. I think the points intended are: 1) LIN-28 may act at that translational level. 2) LIN-28 may not regulate many of the identified transcripts; here I feel "not regulated" is different than "functionally dispensable". Also, a third possibility here is that many of the identified binding sites are false positives. (It's interesting that lin-46 is one of the most significant hits, suggesting that drawing a much more stringent significance cutoff could slightly change the interpretation of the RIP vs RNAseq.)

We have rewritten this section for greater clarity, new ll. 159-166.

2) Related to the point above, the fact that the LIN-28 large tag causes moderate LIN-28 loss of function should be mentioned earlier and more often. In discussing the RIP results, it seems relevant to mention that LIN-28 may not be acting in a completely wild type manner, so the binding events may also be somewhat skewed compared to the ground truth.

We now mention this effect of tagging on LIN-28 when we first introduce the strain for use in RIP-seq (ll. 96-97)

3) Lines 308-309: "The precocious alae 308 synthesis is fully suppressed by loss of either lin-46 or let-7, as neither lin-28(0); lin-46(0) nor lin-28(0); let-7(0) double mutant animals exhibit any alae after the third molt (Fig. 3A)." 
This appears to differ from the data; six percent of lin-28(0); let-7(0) double mutants have alae.

We now clarify that both alleles “strongly” suppress the precocious alae phenotype (ll. 2011-213).

4) In Figure 3A, an additional column regarding the type/extent of alae observed in M4 would be helpful, especially to understand the text about the double and triple mutants.

We have addressed this point (mirroring a similar request from Reviewer #1, minor comment 4) by adding the new Supplementary Figure Fig. S4, which lists the type/extent of alae for the different genotypes.

5) Lines 413-414: "suggesting that the four GGAG motifs in the putative LIN28 binding site are not required for let-7 repression in vivo." 
Although mentioned in the discussion, it would be good to also mention in this position that one possible explanation for the lack of derepression is redundancy of the mutated motifs with other motifs in the transcript.

We now explicitly mention the possibility of binding site redundancy (l. 287).

Reviewer #3 (Significance (Required)): 

General assessment:This work is a very careful genetic dissection of LIN-28's role in the heterochronic pathway and its functional targets. Further supported by molecular data, the epistasis experiments using new genetic tools lend support to a streamlined model of the pathway in which LIN-46 and let-7 derepression drive all of the observed lin-28 null phenotypes. This work also raises the question of how LIN-28 achieves such specificity in regulating lin-46 and let-7 despite evidence of it binding many more mRNA targets. (Are RNA immunoprecipitation experiments generally rife with false positive binding sites? Are the other LIN-28 binding sites mediating translational repression?) The new observation of the role of this pathway in developmental tempo is particularly astute and exciting. As the authors point out, the implication of these genes in tempo will lead to a greater understanding of the relationship of developmental and chronological time in the long term. 
Generally, the conclusions in the manuscript are well supported by the experiments. 
A few very minor changes to the presentation are suggested. 


Audience: All those interested in RNA binding proteins would be interested in this work because of its careful dissection of phenotypically impactful targets compared to empirically-identified biochemical targets. 
This work would also be of great interest to developmental biologists, especially those interested in developmental timing and/or oscillatory gene expression.

We thank the reviewer for highlighting the broad relevance of our work.
