# Supplementary material for: let-7 miRNA and lin-46 mRNA are essential targets of the LIN28 RNA-binding protein in Caenorhabditis elegans developmental timing regulation
Source: PLoS Biol. 2026 Aug 4;24(8):e3003892. doi: 10.1371/journal.pbio.3003892 (PMC13436749; doi:10.1371/journal.pbio.3003892)
Supplement: Supplementary file 13 [file pbio.3003892.s017.docx]

Reviewer remarks:


Reviewer #1: The edits have improved the paper. The information about the deletion identified in the Ambros strain is useful to know and it is good to see that included in the methods. I agree that given the circumstances that is the best placement for it. I have only one remaining comment and it concerns the question of null alleles.  Since, as the authors state, they were not able to show that the let-7 allele is null, this allele cannot be called "let-7(0)" in the paper. Below I will elaborate on this statement, but from a practical standpoint, the authors should eliminate the claim that they are using a null allele and change "let-7(0)" to "let-7(-)" or similar throughout the paper.

The concern with using non-null alleles for epistasis experiments is that any remaining gene product may still function and that function may be amplified when a negative regulator is removed. When the potential contribution of the remaining gene activity is ignored, that can result in incorrect interpretations and has led to erroneous genetic pathways in past published literature. Thus, if the let-7 allele is non-null, the difference in phenotype between let-7(-) and lin-28(0); let-7(-) strains may be due, at least in part, to increased let-7 activity in the lin-28(0) background. However, I take the authors' point that in this case, since the lin-46(0); let-7(-) double mutant completely suppresses the lin-28(0) phenotypes and loss of lin-28 does not affect the lin-46(0); let-7(-) phenotype, the lack of a null allele does not prohibit the argument that lin-46 and let-7 together appear to account for the lin-28 phenotypes. Therefore, while I still think that using a null
allele is best, I agree that it is not required for the specific point the authors are making. I agree with not using the classic mn112 allele for the reasons mentioned, and it is indeed unfortunate that the authors did not create a true null allele at the outset. My opinion is that the current allele can be used with the appropriate wording.

It is not clear what basis the authors have for saying that the allele is "most likely" a null (new line 190) when in fact the evidence seems to be the opposite. Specifically, the qPCR data show much reduced but still detectable levels of mature let-7 at both timepoints. I am not sure if the authors are arguing that very low levels of gene product is equivalent to a null or if they believe that they are seeing non-specific amplification in their let-7(xe150) allele. A null means no gene product. To quote the textbook by Hawley and Walker "Nullomorphs (or amorphs) are mutants with no remaining gene function, i.e., they produce no functional gene product." In contrast, low levels of gene product would be a hypomorphic allele. If the authors would like to argue that they do have a null and the amplification they are seeing is nonspecific, they could strengthen their argument by comparing lin-28(0); let-7(xe150) vs. let-7(xe150) to determine if let-7 levels increase in a lin-28
background. However, that experiment is not necessary if the wording is changed.

The other arguments presented are not convincing to me. Since mature let-7 is produced from the primary transcript and the primary transcript is typically much less abundant than the mature miRNA, the inability to detect pri-let-7 at one time point does not negate the ability to detect mature let-7 at that same timepoint. In addition, the strength of the xe150 phenotype indicates that let-7 activity is below the threshold needed for wild-type function but does not provide any information regarding whether the allele is null. There are many examples of hypomorphic alleles that have the same phenotype as null alleles, at least in a wild-type background. Use of those hypomorphic alleles has in some cases given rise to erroneous conclusions when used for epistasis. Use of correct terminology is important both for interpretation in this paper and as precedent for any future work using this allele.

We thank the Reviewer for clarifying their point and have followed their recommendation to change “let-7(0)” to “let-7(-)” throughout the paper.


Reviewer #2 (Amy E. Pasquinelli, identifies herself): The manuscript by Brunner et al., investigates the molecular and biologically relevant targets of the LIN-28 RNA binding protein in C. elegans. While LIN-28 has been shown to binds thousands of RNAs, including the let-7 miRNA primary transcript, the regulatory role and biological importance of this extensive network has been unclear. Here the authors use molecular approaches to investigate the RNAs bound and regulated by LIN-28 and genetic experiments to determine which targets are important for C. elegans development and viability. Remarkably, only two genes, lin-46 and let-7, emerged as functionally essential targets. It was previously reported that the lin-46 mRNA is bound and translationally repressed by LIN-28 protein and processing of the let-7 primary transcript is blocked by LIN-28. Using careful and thorough molecular and phenotypic assays of genetic mutants, the authors now convincingly show that lin-46 and
let-7 are the only relevant targets of LIN-28, at least under standard growth conditions. Furthermore, they reveal a new defect, slowed progression through larval stages, caused by loss of lin-28 and regulation of its targets. Additionally, this work expands our understanding of lin-46 regulation by LIN-28, showing that RNA destabilization is involved as well as likely more binding sites than the previously identified site in the 5'UTR. They also directly tested the predicted LIN-28 binding site in the let-7 primary transcript and found that its removal is not sufficient to reproduce the level of mis-regulation seen in the lin-28 loss of function strain. While the authors do not attempt to map the presumably additional LIN-28 regulatory sites in lin-46 or let-7 these studies do demonstrate that recognition of these targets by LIN-28 is not solved. Overall, the experiments are rigorous, well controlled and the results support the conclusions. The paper is scholarly and
clearly written with attention to referencing prior studies and limitations of the study.
This paper is significant because it deeply investigates the biologically important targets of the well conserved LIN-28 RNA binding protein. It provides a conceptual advance that pinpoints key genes that require regulation by LIN-28, despite its extensive binding repertoire. While it is surprising that only 2 targets emerged as relevant for the role of lin-28 in regulating the identity and pace of larval development, this finding may inform on the function of LIN28 in mammalian systems. Regulation of let-7 biogenesis is a conserved function of LIN28 but a consistent role in regulating the thousands mRNAs it binds in mammalian cells has been lacking. As mammalian LIN28 has roles in controlling puberty onset and cancer development, a better understanding of its biologically relevant targets will be of interest to a broad audience in the RNA and development fields.

We thank Dr. Pasquinelli for the endorsement of our work.
