# Supplementary material for: let-7 miRNA and lin-46 mRNA are essential targets of the LIN28 RNA-binding protein in Caenorhabditis elegans developmental timing regulation
Source: PLoS Biol. 2026 Aug 4;24(8):e3003892. doi: 10.1371/journal.pbio.3003892 (PMC13436749; doi:10.1371/journal.pbio.3003892)
Supplement: Peer review history

# Author comment: Author response to Decision Letter 0

**DOI:** 10.1371/journal.pbio.3003892.r001

22 Apr 2026

Attachment Submitted filename: 165355_1_rebuttal_3720009_td47m1.docx

# Editor report: Decision Letter 0

**DOI:** 10.1371/journal.pbio.3003892.r002

**Authors:** Richard Hodge

5 May 2026

Dear Dr Großhans,

Thank you for submitting your revised manuscript from Review Commons entitled "let-7 miRNA and lin-46 mRNA are the two essential targets of the LIN28 RNA-binding protein in developmental timing" for consideration as a Research Article by PLOS Biology. Please accept my sincere apologies for the delay in getting back to you as we consulted with an academic editor about your submission.

Your manuscript has now been evaluated by the PLOS Biology editorial staff, as well as by an academic editor with relevant expertise, and I am writing to let you know that we would like to send your revision out for re-review by the previous reviewers at Review Commons.

However, before we can send your manuscript to reviewers, we need you to complete your submission by providing the metadata that is required for full assessment. To this end, please login to Editorial Manager where you will find the paper in the 'Submissions Needing Revisions' folder on your homepage. Please click 'Revise Submission' from the Action Links and complete all additional questions in the submission questionnaire.

Once your full submission is complete, your paper will undergo a series of checks in preparation for peer review. After your manuscript has passed the checks it will be sent out for review. To provide the metadata for your submission, please Login to Editorial Manager (https://www.editorialmanager.com/pbiology) within two working days, i.e. by May 07 2026 11:59PM.

During the process of completing your manuscript submission, you will be invited to opt-in to posting your pre-review manuscript as a bioRxiv preprint. Visit http://journals.plos.org/plosbiology/s/preprints for full details. If you consent to posting your current manuscript as a preprint, please upload a single Preprint PDF.

Feel free to email us at plosbiology@plos.org if you have any queries relating to your submission.

Kind regards,

Richard

Richard Hodge, PhD

Senior Editor, PLOS Biology

rhodge@plos.org

PLOS

Empowering researchers to transform science

Carlyle House, Carlyle Road, Cambridge, CB4 3DN, United Kingdom

California (U.S.) corporation #C2354500, based in San Francisco

# Author comment: Author response to Decision Letter 1

**DOI:** 10.1371/journal.pbio.3003892.r003

7 May 2026

Attachment Submitted filename: 165355_1_rebuttal_3720009_td47m1_auresp_1.docx

# Peer review: Decision Letter 1

**DOI:** 10.1371/journal.pbio.3003892.r004

**Authors:** Richard Hodge

1 Jun 2026

Dear Dr Großhans,

Thank you for your patience while we considered your revised manuscript "let-7 miRNA and lin-46 mRNA are the two essential targets of the LIN28 RNA-binding protein in developmental timing" for publication as a Research Article at PLOS Biology. Please accept my apologies for the delays that you have experienced during the peer review process. This revised version of your manuscript has been evaluated by the PLOS Biology editors, the Academic Editor and the original reviewers at Review Commons. Please note that Reviewer #3 did re-review the manuscript but did not provide any specific comments.

Based on the reviews, we are likely to accept this manuscript for publication, provided you satisfactorily address the remaining points raised by Reviewer #1. In addition, please make sure to address the following data and other policy-related requests that I have provided below (A-H):

(A)We routinely suggest changes to titles to ensure maximum accessibility for a broad, non-specialist readership. In this case, we would suggest a couple of minor edits to the title for flow and additional information, as follows. Please ensure you change both the manuscript file and the online submission system, as they need to match for final acceptance:

"let-7 miRNA and lin-46 mRNA are essential targets of the LIN28 RNA-binding protein in C. elegans developmental timing regulation"

(B) You may be aware of the PLOS Data Policy, which requires that all data be made available without restriction: http://journals.plos.org/plosbiology/s/data-availability. For more information, please also see this editorial: http://dx.doi.org/10.1371/journal.pbio.1001797

Note that we do not require all raw data. Rather, we ask that all individual quantitative observations that underlie the data summarized in the figures and results of your paper be made available in one of the following forms:

-Supplementary files (e.g., excel). Please ensure that all data files are uploaded as 'Supporting Information' and are invariably referred to (in the manuscript, figure legends, and the Description field when uploading your files) using the following format verbatim: S1 Data, S2 Data, etc. Multiple panels of a single or even several figures can be included as multiple sheets in one excel file that is saved using exactly the following convention: S1_Data.xlsx (using an underscore).

-Deposition in a publicly available repository. Please also provide the accession code or a reviewer link so that we may view your data before publication.

Regardless of the method selected, please ensure that you provide the individual numerical values that underlie the summary data displayed in the following figure panels as they are essential for readers to assess your analysis and to reproduce it:

Figure 1B-G, 2B, 3A, 4A-B, 4D, 5A, 6A-D, 7A, 7C-F, 8A-D, S1A-B, S2, S3B-D, S6B, S7A-D, S8

NOTE: the numerical data provided should include all replicates AND the way in which the plotted mean and errors were derived (it should not present only the mean/average values).

(C) Thank you for providing the RNA-sequencing data in the GEO database (GSE315957). However, we note that the data is currently on hold for release. We ask that you please make the data publicly available at this stage before publication. In addition, I would be grateful if you could clarify whether the RIP-seq dataset is included in this deposition?

(D) Please also ensure that each of the relevant figure legends in your manuscript include information on *WHERE THE UNDERLYING DATA CAN BE FOUND*, and ensure your supplemental data file/s has a legend.

(E) We require the original, uncropped and minimally adjusted images supporting all blot and gel results reported in the following Figures:

Figure S6A

We will require these files before a manuscript can be accepted so please prepare and upload them now. Please carefully read our guidelines for how to prepare and upload this data: https://journals.plos.org/plosbiology/s/figures#loc-blot-and-gel-reporting-requirements.

(F) Per journal policy, if you have generated any custom code during the course of this investigation, please make it available without restrictions. Please ensure that the code is sufficiently well documented and reusable, and that your Data Statement in the Editorial Manager submission system accurately describes where your code can be found. More information on our Code Policy, what and how to share can be found here: https://journals.plos.org/plosbiology/s/code-availability

Please note that we cannot accept sole deposition of code in GitHub, as this could be changed after publication. However, you can archive this version of your publicly available GitHub code to Zenodo. Once you do this, it will generate a DOI number, which you will need to provide in the Data Accessibility Statement (you are welcome to also provide the GitHub access information). See the process for doing this here: https://docs.github.com/en/repositories/archiving-a-github-repository/referencing-and-citing-content

(G) Please ensure that you are using best practice for statistical reporting and data presentation. These are our guidelines https://journals.plos.org/plosbiology/s/best-practices-in-research-reporting#loc-statistical-reporting and a useful resource on data presentation https://journals.plos.org/plosbiology/article?id=10.1371/journal.pbio.1002128

- If you are reporting experiments where n ≤ 5, please plot each individual data point.

(H) Please ensure that your Data Statement in the submission system accurately describes where your data can be found and is in final format, as it will be published as written there.

------------------------------------------------------------------------

As you address these items, please take this last chance to review your reference list to ensure that it is complete and correct. If you have cited papers that have been retracted, please include the rationale for doing so in the manuscript text, or remove these references and replace them with relevant current references. Any changes to the reference list should be mentioned in the cover letter that accompanies your revised manuscript.

In addition to these revisions, you may need to complete some formatting changes, which you will receive in a follow up email. A member of our team will be in touch with a set of requests shortly. If you do not receive a separate email within a few days, please assume that checks have been completed, and no additional changes are required.

We expect to receive your revised manuscript within two weeks.

To submit your revision, please go to https://www.editorialmanager.com/pbiology/ and log in as an Author. Click the link labelled 'Submissions Needing Revision' to find your submission record. Your revised submission must include the following:

- a cover letter that should detail your responses to any editorial requests, if applicable, and whether changes have been made to the reference list

- a Response to Reviewers file that provides a detailed response to the reviewers' comments (if applicable, if not applicable please do not delete your existing 'Response to Reviewers' file.)

- a track-changes file indicating any changes that you have made to the manuscript.

NOTE: If Supporting Information files are included with your article, note that these are not copyedited and will be published as they are submitted. Please ensure that these files are legible and of high quality (at least 300 dpi) in an easily accessible file format. For this reason, please be aware that any references listed in an SI file will not be indexed. For more information, see our Supporting Information guidelines:

https://journals.plos.org/plosbiology/s/supporting-information

*Published Peer Review History*

Please note that you may have the opportunity to make the peer review history publicly available. The record will include editor decision letters (with reviews) and your responses to reviewer comments. If eligible, we will contact you to opt in or out. Please see here for more details:

https://plos.org/published-peer-review-history/

*Press*

Should you, your institution's press office or the journal office choose to press release your paper, please ensure you have opted out of Early Article Posting on the submission form. We ask that you notify us as soon as possible if you or your institution is planning to press release the article.

*Protocols deposition*

To enhance the reproducibility of your results, we recommend that if applicable you deposit your laboratory protocols in protocols.io, where a protocol can be assigned its own identifier (DOI) such that it can be cited independently in the future. Additionally, PLOS ONE offers an option for publishing peer-reviewed Lab Protocol articles, which describe protocols hosted on protocols.io. Read more information on sharing protocols at https://plos.org/protocols?utm_medium=editorial-email&utm_source=authorletters&utm_campaign=protocols

Please do not hesitate to contact me should you have any questions.

Best regards,

Richard

Richard Hodge, PhD

Senior Editor, PLOS Biology

rhodge@plos.org

------------------------------------------------------------------------

Reviewer remarks:

Reviewer #1: The edits have improved the paper. The information about the deletion identified in the Ambros strain is useful to know and it is good to see that included in the methods. I agree that given the circumstances that is the best placement for it. I have only one remaining comment and it concerns the question of null alleles. Since, as the authors state, they were not able to show that the let-7 allele is null, this allele cannot be called "let-7(0)" in the paper. Below I will elaborate on this statement, but from a practical standpoint, the authors should eliminate the claim that they are using a null allele and change "let-7(0)" to "let-7(-)" or similar throughout the paper.

The concern with using non-null alleles for epistasis experiments is that any remaining gene product may still function and that function may be amplified when a negative regulator is removed. When the potential contribution of the remaining gene activity is ignored, that can result in incorrect interpretations and has led to erroneous genetic pathways in past published literature. Thus, if the let-7 allele is non-null, the difference in phenotype between let-7(-) and lin-28(0); let-7(-) strains may be due, at least in part, to increased let-7 activity in the lin-28(0) background. However, I take the authors' point that in this case, since the lin-46(0); let-7(-) double mutant completely suppresses the lin-28(0) phenotypes and loss of lin-28 does not affect the lin-46(0); let-7(-) phenotype, the lack of a null allele does not prohibit the argument that lin-46 and let-7 together appear to account for the lin-28 phenotypes. Therefore, while I still think that using a null allele is best, I agree that it is not required for the specific point the authors are making. I agree with not using the classic mn112 allele for the reasons mentioned, and it is indeed unfortunate that the authors did not create a true null allele at the outset. My opinion is that the current allele can be used with the appropriate wording.

It is not clear what basis the authors have for saying that the allele is "most likely" a null (new line 190) when in fact the evidence seems to be the opposite. Specifically, the qPCR data show much reduced but still detectable levels of mature let-7 at both timepoints. I am not sure if the authors are arguing that very low levels of gene product is equivalent to a null or if they believe that they are seeing non-specific amplification in their let-7(xe150) allele. A null means no gene product. To quote the textbook by Hawley and Walker "Nullomorphs (or amorphs) are mutants with no remaining gene function, i.e., they produce no functional gene product." In contrast, low levels of gene product would be a hypomorphic allele. If the authors would like to argue that they do have a null and the amplification they are seeing is nonspecific, they could strengthen their argument by comparing lin-28(0); let-7(xe150) vs. let-7(xe150) to determine if let-7 levels increase in a lin-28 background. However, that experiment is not necessary if the wording is changed.

The other arguments presented are not convincing to me. Since mature let-7 is produced from the primary transcript and the primary transcript is typically much less abundant than the mature miRNA, the inability to detect pri-let-7 at one time point does not negate the ability to detect mature let-7 at that same timepoint. In addition, the strength of the xe150 phenotype indicates that let-7 activity is below the threshold needed for wild-type function but does not provide any information regarding whether the allele is null. There are many examples of hypomorphic alleles that have the same phenotype as null alleles, at least in a wild-type background. Use of those hypomorphic alleles has in some cases given rise to erroneous conclusions when used for epistasis. Use of correct terminology is important both for interpretation in this paper and as precedent for any future work using this allele.

Reviewer #2 (Amy E. Pasquinelli, identifies herself): The manuscript by Brunner et al., investigates the molecular and biologically relevant targets of the LIN-28 RNA binding protein in C. elegans. While LIN-28 has been shown to binds thousands of RNAs, including the let-7 miRNA primary transcript, the regulatory role and biological importance of this extensive network has been unclear. Here the authors use molecular approaches to investigate the RNAs bound and regulated by LIN-28 and genetic experiments to determine which targets are important for C. elegans development and viability. Remarkably, only two genes, lin-46 and let-7, emerged as functionally essential targets. It was previously reported that the lin-46 mRNA is bound and translationally repressed by LIN-28 protein and processing of the let-7 primary transcript is blocked by LIN-28. Using careful and thorough molecular and phenotypic assays of genetic mutants, the authors now convincingly show that lin-46 and let-7 are the only relevant targets of LIN-28, at least under standard growth conditions. Furthermore, they reveal a new defect, slowed progression through larval stages, caused by loss of lin-28 and regulation of its targets. Additionally, this work expands our understanding of lin-46 regulation by LIN-28, showing that RNA destabilization is involved as well as likely more binding sites than the previously identified site in the 5'UTR. They also directly tested the predicted LIN-28 binding site in the let-7 primary transcript and found that its removal is not sufficient to reproduce the level of mis-regulation seen in the lin-28 loss of function strain. While the authors do not attempt to map the presumably additional LIN-28 regulatory sites in lin-46 or let-7 these studies do demonstrate that recognition of these targets by LIN-28 is not solved. Overall, the experiments are rigorous, well controlled and the results support the conclusions. The paper is scholarly and clearly written with attention to referencing prior studies and limitations of the study.

This paper is significant because it deeply investigates the biologically important targets of the well conserved LIN-28 RNA binding protein. It provides a conceptual advance that pinpoints key genes that require regulation by LIN-28, despite its extensive binding repertoire. While it is surprising that only 2 targets emerged as relevant for the role of lin-28 in regulating the identity and pace of larval development, this finding may inform on the function of LIN28 in mammalian systems. Regulation of let-7 biogenesis is a conserved function of LIN28 but a consistent role in regulating the thousands mRNAs it binds in mammalian cells has been lacking. As mammalian LIN28 has roles in controlling puberty onset and cancer development, a better understanding of its biologically relevant targets will be of interest to a broad audience in the RNA and development fields.

# Author comment: Author response to Decision Letter 2

**DOI:** 10.1371/journal.pbio.3003892.r005

12 Jun 2026

Attachment Submitted filename: Point-by-point-response-to-reviewers.docx

# Editor report: Decision Letter 2

**DOI:** 10.1371/journal.pbio.3003892.r006

**Authors:** Richard Hodge

22 Jun 2026

Dear Helge,

On behalf of my colleagues and the Academic Editor, Amy Pasquinelli, I am pleased to say that we can accept your manuscript for publication, provided you address any remaining formatting and reporting issues. These will be detailed in an email you should receive within 2-3 business days from our colleagues in the journal operations team; no action is required from you until then. Please note that we will not be able to formally accept your manuscript and schedule it for publication until you have completed any requested changes.

In addition, I note that there is a reference to (Katic et al, in preparation) on line 860 in the Discussion section. I would be grateful if you could please update and provide the citation for the published preprint during the production process.

Please take a minute to log into Editorial Manager at http://www.editorialmanager.com/pbiology/, click the "Update My Information" link at the top of the page, and update your user information to ensure an efficient production process.

PRESS

We frequently collaborate with press offices. If your institution or institutions have a press office, please notify them about your upcoming paper at this point, to enable them to help maximise its impact. If the press office is planning to promote your findings, we would be grateful if they could coordinate with biologypress@plos.org. If you have previously opted in to the early version process, we ask that you notify us immediately of any press plans so that we may opt out on your behalf.

We also ask that you take this opportunity to read our Embargo Policy regarding the discussion, promotion and media coverage of work that is yet to be published by PLOS. As your manuscript is not yet published, it is bound by the conditions of our Embargo Policy. Please be aware that this policy is in place both to ensure that any press coverage of your article is fully substantiated and to provide a direct link between such coverage and the published work. For full details of our Embargo Policy, please visit http://www.plos.org/about/media-inquiries/embargo-policy/.

Thank you again for choosing PLOS Biology for publication and supporting Open Access publishing. We look forward to publishing your study.

Best wishes,

Richard

Richard Hodge, PhD

Senior Editor, PLOS Biology

rhodge@plos.org

PLOS

Empowering researchers to transform science

Carlyle House, Carlyle Road, Cambridge, CB4 3DN, United Kingdom

California (U.S.) corporation #C2354500, based in San Francisco
